# In the face of change: Which coping strategies predict better psychosocial outcomes in face transplant recipients?

**DOI:** 10.3389/fpsyg.2022.995222

**Published:** 2022-11-17

**Authors:** Marie-Christine Nizzi, Bohdan Pomahac

**Affiliations:** ^1^Dartmouth College, Hanover, MD, United States; ^2^UCLA Health System, Los Angeles, CA, United States; ^3^Chapman University, Los Angeles, CA, United States; ^4^Yale University, New Haven, CT, United States

**Keywords:** coping, vascularized composite tissue allotransplantation, quality of life, Selfesteem, depression, psychosocial outcomes, face transplant, outcomes

## Abstract

**Objectives:**

Face transplantation aims to improve patients’ quality of life and psychosocial functioning in patients with a disfiguring injury. With 40 cases worldwide, little is known about coping strategies predicting resilient outcomes.

**Design:**

Six patients followed in Boston, completed the Brief COPE (Carver, 1997) along with validated measures of depression, self-esteem, and quality of life – every 3 months during the first year post-transplant and every 6 months thereafter, up to 36 months post-transplant.

**Analyses:**

Due to sample size and distribution of the data, nonparametric tests were used to characterize the relation of coping strategies with psychosocial outcomes.

**Results:**

As expected, active coping strategies were associated with better mental health pre-transplant, while avoidant coping strategies were associated with poorer mental health. Patients using support-based strategies reported better mental health at baseline. Post-transplant, the pattern reversed such that avoidant strategies appeared protective, when looking at mental health trajectories over 18 months. Importantly, trends identified during the first 18 months matched the trajectories of all patients with existing data up to 36 months post-transplant, for all outcomes measured.

**Conclusion:**

Different coping strategies support optimal outcomes in the pre-versus post-transplant phases. Pre-transplant data may better inform interventions supporting mental health of transplant candidates than predict post-transplant behavior. Early post-transplant data seems to provide promising insight in long term psychosocial outcomes.

**Clinical implications:**

Our data stresses the need for pre-transplant assessment of coping and post-transplant coping training. Research aiming to optimize post-transplant psychosocial outcomes should consider coping as a promising target for intervention.

## Introduction

“Some things are in our control and others not. Things in our control are opinion, pursuit, desire, aversion, and, in a word, whatever are our own actions. Things not in our control are body, property, reputation, command, and, in one word, whatever are not our own actions” Epictetus, *The Enchiridion*, 135 ACE.

“When life gives you lemons, make lemonade!” Various origins.

Philosophers and popular wisdom agree: the way we react to adversity can help us come out of it on top. But what is the recipe: what are the best ways to cope? This question is particularly important in the context of experimental procedures, where the psychological and surgical teams advising potential candidates have little to no data available to inform their decisions regarding patient selection and treatment recommendations. Vascularized composite allotransplantation (VCA) comprises surgeries such as face transplant, upper-extremity transplant, abdominal wall transplant, etc. Among them, face transplantation may soon become standard of care and be offered much more widely than it has been so far. Medical teams are looking for guidelines to inform patient selection. This makes it particularly important to identify which coping strategies at baseline are associated with better psychosocial outcomes post-transplant.

Face transplantation is an innovative surgical procedure aiming to restore appearance and function in patients with a history of severe facial trauma. Since the world first case in France in 2005, more than 40 patients have received partial or full face transplants around the world (Oser et al., 2018; Tasigiorgos et al., 2019; Kauke et al., 2021). Yet little is known about the psychological traits fostering successful adjustment post-transplant (Nizzi et al., 2017) and patient reported outcomes about quality of life after drastic physical changes are often received with skepticism (Nizzi, 2021). In this longitudinal study of six face-transplant recipients, we investigated which coping strategies predicted better psychosocial outcomes in terms of self-esteem, depression, and quality of life, within the first 18 months post-transplant, and up to 36 months for the first three recipients.

Several measures of coping have been developed over the past 50 years (Billings and Moos, 1981; Folkman and Lazarus, 1985; Endler and Parker, 1990). We used the Brief COPE (Carver et al., 1989; Carver, 1997). This 28-item measure evaluates 14 coping strategies – each assessed by 2 items in the Lickert-scale – and has demonstrated good reliability/validity in similar populations, such as hospitalized patients with burn injury (Amoyal et al., 2011). Factor analyses have yielded inconsistent findings. However, one constant seems to be the distinction between active-approach coping and avoidant coping (Lawrence and Fauerbach, 2003; Prado et al., 2004). Strategies related to active coping often include acceptance, positive reframing, and a proactive problem-solving attitude. Strategies related to avoidant coping include denial, self-blame, and behavioral disengagement. New factors added to those identified in the 1989 study by Carver and colleagues include support seeking, self-distraction, venting, humor, and religion (Carver, 1997).

The distinction between active and avoidant coping is supported by an extensive literature, both in adolescents and adults, and across a variety of conditions (Canada et al., 2006; Frydenberg and Lewis, 2009; Montel et al., 2012; Asuzu and Elumelu, 2013; Barendregt et al., 2015). We first hypothesized that active coping strategies – such as acceptance and positive reframing – would be associated with a higher self-esteem, higher quality of life, and lower depression levels. Conversely, we hypothesized that items pertaining to avoidant coping – such as denial, self-blame, substance use and behavioral disengagement – would be associated with higher depression scores, lower self-esteem, and lower quality of life scores. Our second hypothesis was derived from clinical practice. When assessing pre-surgical candidates, it is commonly considered to be a good protective factor when a candidate has a strong social support system to rely on. We predicted that support-based strategies would be associated with positive mental health outcomes.

Given the small sample and worldwide population size, our goal in this study is to provide an initial description of coping strategies in relation to key mental health outcomes in face-transplant recipients pre-and post-transplant. Our approach is that of a pioneer study, where it is very difficult to access more cases. We expect our result to be meaningful, but they should be interpreted with caution and the ambition to shed initial light on this under-researched field rather than predicting or generalizing findings. We expect our findings to contribute to informing the direction for future studies.

## Materials and methods

### Sample

Six face transplant recipients participated in this study (2 females, 25 to 57 years old at time of transplantation, mean age = 38). All patients were followed at Brigham and Women’s Hospital in Boston at the time of data collection. For a detailed presentation of each case in the cohort, please refer to the cohort description (Oser et al., 2018).

### Procedure

Patients completed both coping and outcome measures at baseline prior to their facial transplantation. Patients then completed outcome measures every 3 months during the first year, and every 6 months thereafter.

### Measures

#### Coping

The brief form of the COPE Inventory (Carver, 1997) is used to characterize the strategies one uses to cope with their stress. The 28-item Likert scale assesses 14 different coping behaviors: self-distraction, active coping, denial, substance use, emotional support, instrumental support, behavioral disengagement, venting, positive reframing, planning, humor, acceptance, religion, and self-blame. The 4-point Likert scale assesses frequency, with higher scores indicating that the respondent reported using the coping strategy more frequently. A sum score for each of the 14 scales can be derived by summing the individual scores for each of the two items of the scale. The Brief COPE does not provide an overall score, cluster-scores, or cut-off scores. It is recommended to define adaptive and maladaptive composites based on previous literature in the relevant population or second-order factors derived from each sample, and to analyze each scale separately to characterize its relation to other variables of interest.^1^ The psychometric properties of this scale have been evaluated in hospitalized patients with disfiguring injuries and have demonstrated good reliability, construct validity, and factor structure (Amoyal et al., 2011). To date, the Brief-COPE has been translated in several languages, including French, German, Greek, Korean, and Spanish.

#### Self esteem

Rosenberg’s Self Esteem Scale (RSES (Rosenberg, 1965) has received more psychometric validation than any other measure of self-esteem (Robins et al., 2001). Scores on this 10-item Likert scale range from 10 to 40, with a higher score indicating a higher level of self-esteem. Whilst originally developed with adolescent populations, the scale has widely used across adult populations (Robins et al., 2001) and has good internal consistency specifically with patients suffering from disfiguring injuries (Nicolosi et al., 2013).

#### Depression

The Center for Epidemiologic Studies Depression Scale (CES-D) is a short self-report scale designed to measure depressive symptomatology. It consists of a validated list of 20 symptoms, each rated by responders on a scale of 0 to 3 according to its prevalence during the past week (0 = rarely or none of the time, 1 = some or little of the time, 2 = moderately or much of the time, 3 = most or all the time, Radloff, 1977; Lewinsohn et al., 1997). The total score, ranging from 0 to 60 (clinical cut-off at 16), indicates the sum prevalence of depressive symptoms, and corresponds with high sensitivity and specificity to the overall risk of clinical depression (Lewinsohn et al., 1997).

#### Health related quality of life

The EQ-5D was administered to assess health-related quality of life using a visual analogue scale to rate current health state along a continuous scale from 0 (“worst imaginable health state”) to 100 (“best imaginable health state”). The measure has shown good reliability with a range of clinical populations (Cleemput et al., 2004) and strong construct validity in patients with severe skin injuries (Oster et al., 2009; Shaw et al., 2005).

### Statistical analyses

Statistical analyses were completed using the R software, version 3.2.2. Significance level was set at 0.05. Due to sample size and distribution of the data, nonparametric tests were used to calculate correlations first within, then across subjects. Bonferroni correction was applied for multiple comparisons.

Within subjects, we explored the relations between outcome measures, based on each patient’s scores over the first 18 months post-transplant, using Spearman correlation coefficients.

**Figure 1:**
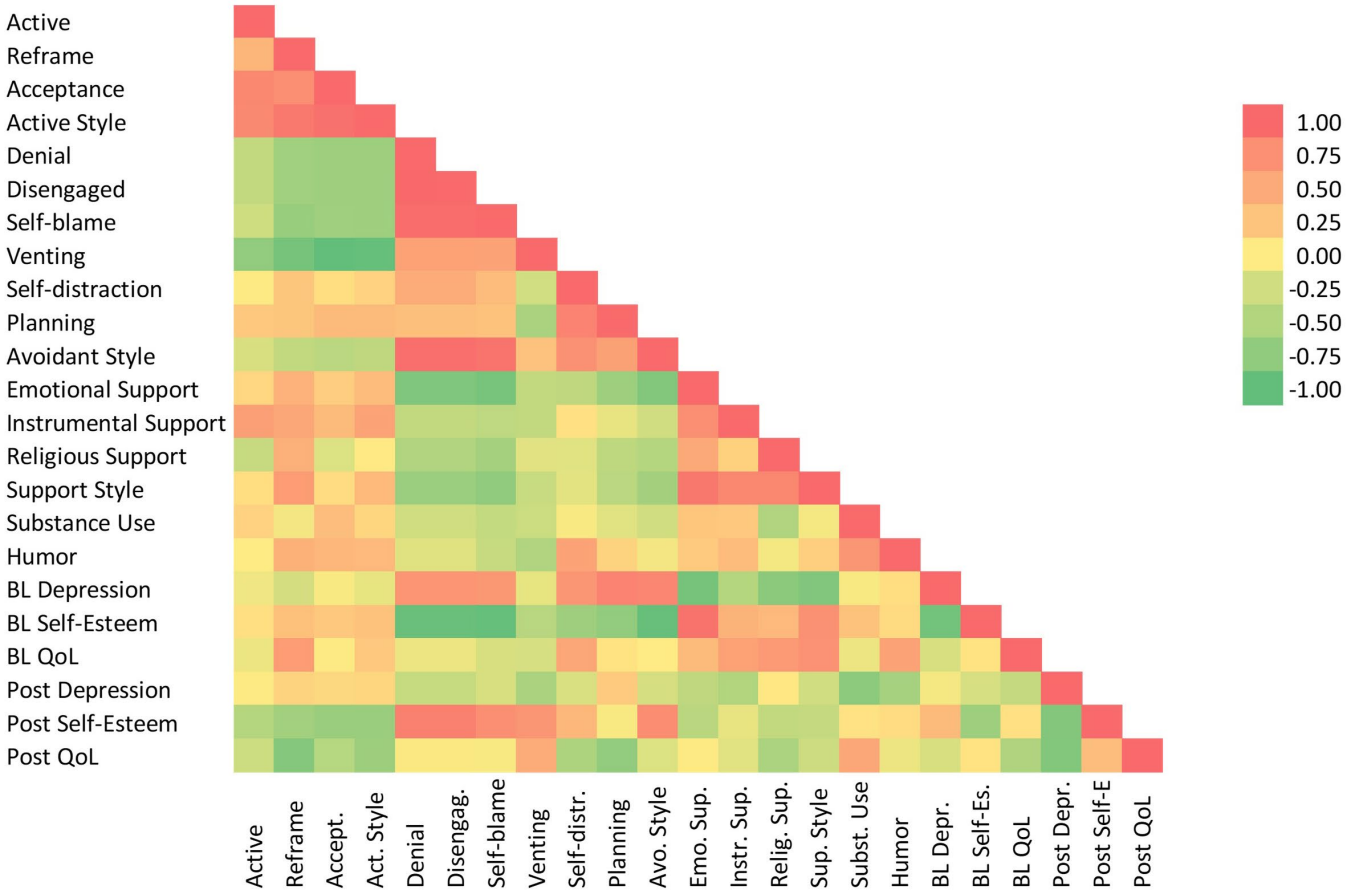
Heat map.

Across subjects, we explored the relations between the 14 coping strategies using Spearman correlation coefficients (lines 1–17 in Figure 1). We then explored the relation between each coping strategy and each psychosocial measure, at baseline (lines 18–20).

For post-transplant outcome analyses, we computed each patient’s average score for post-transplant outcome measures, then correlated this average score with their baseline coping scores (lines 21–23). In half of our sample, we also collected data from 18 to 36 months post-transplant. For these patients, we fit a linear regression line for each outcome measure to determine their outcome trajectories based on data from baseline to 18 months. Trend projections were then tested in data collected from 18 to 36 months post-transplant, using visual analysis of mental health trajectories as well as a comparison of slopes obtained in the linear regressions.

## Results

At the individual level, five of the six patients showed associations in the expected direction between mental health outcomes. Depression was negatively correlated with self-esteem (= −0.14 to-0.94) and with quality of life (*r* = −0.68 to-0.97), while self-esteem was positively correlated with quality of life (*r* = 0.11 to 0.99). One patient showed an inverted pattern of response, such that depression was positively correlated with self-esteem (*r* = 0.63, *p* < 0.01) and had a similar trend for quality of life (*r* = 0.16, ns), while self-esteem trended towards negative correlation with quality of life (*r* = −0.20, ns). Results for the three mental health outcome measures are thus reported separately.

At the group level for baseline (Figure 1), only positive reframing had a significant association with mental health outcomes among active coping strategies, with QoL (*r* = 0.66, *p* < 0.001) and self-esteem (*r* = 0.41, *p* = 0.05). Acceptance trended with higher self-esteem (*r* = 0.36, ns). Among avoidant strategies, denial, disengagement, and self-blame were all strongly associated with higher depression and lower self-esteem (Figure 1). In our sample, planning seems to have behaved like self-distraction: both were also strongly associated with higher depression (correlations ranging from 0.69 to 0.71, *p* < 0.001) and lower self-esteem (*r* = 0.90 to 0.93, *p* < 0.001). Planning had the highest correlation with depression scores (*r* = 0.82, *p* < 0.001). Self-distraction stood out among avoidant strategies for a moderate positive correlation with QoL.

All three support-based strategies were associated with lower depression (*r* = −0.38 to-0.81), higher self-esteem (*r* = 0.47 to 0.94), and higher QoL (*r* = 0.45 to 0.67). Receiving emotional support from others was strongly protective, followed by finding support in one’s faith. Humor showed a moderate to high positive correlation with QoL (*r* = 0.61, *p* < 0.001).

To look at trends over 18 months post-transplant, a linear regression line was fitted for each patient in each mental health outcome measured. Overall, 2 patients worsened across all outcomes (S1, S5), 2 patients improved across all outcomes (S2, S6), and 2 patients remained stable with mixed patterns (better QoL but worse depression). We found a strong negative correlation both between depression and self-esteem and between depression and QoL, while we found a moderate positive correlation between self-esteem and QoL, all of which went in the expected direction. Comparing pre-and post-outcome measures, depression scores showed a very low positive correlation with each other, while self-esteem and QoL showed moderate negative correlations between baseline assessment and post-transplant trend.

Post-transplant (Figure 1), active coping strategies were associated with worsening self-esteem and QoL trends. Positive reframing and acceptance were associated with decreased self-esteem (*r* = −0.51, *p* < 0.05) and lower QoL (*r* = −0.71, *p* < 0.001). Avoidant strategies like denial, disengagement, and self-blame were all associated with higher self-esteem post-transplant (*r* = 0.75 to 0.84, *p* < 0.001). Venting was also associated with lower depression trend (*r* = −0.46, *p* < 0.05) and higher QoL (*r* = 0.55, *p* < 0.01). Self-distraction and planning continued to be associated with worsening QoL post-transplant. Overall, avoidant strategies correlated with better self-esteem post-transplant.

The three support-based strategies continued to trend towards lower depression but failed to reach significance. Religious support correlated with worsening QoL trends (*r* = −0.45, *p* < 0.05). Humor was associated with lower depression trends (*r* = −0.48, *p* < 0.05).

**Figure 2:**
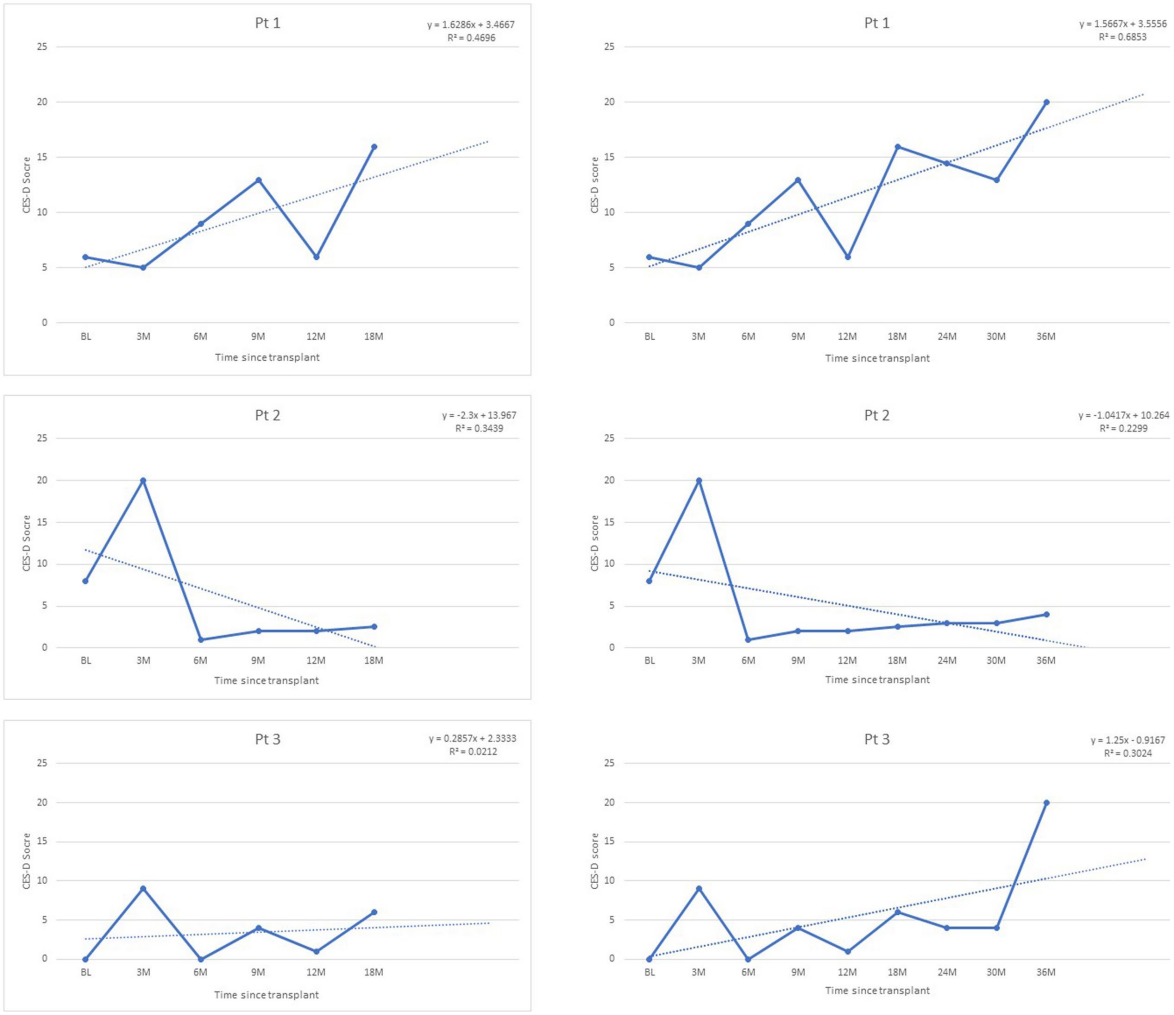
Depression trajectories post-transplant, as measured by CES-D scores, range 0–60 (clinical cut-off >16).

**Figure 3:**
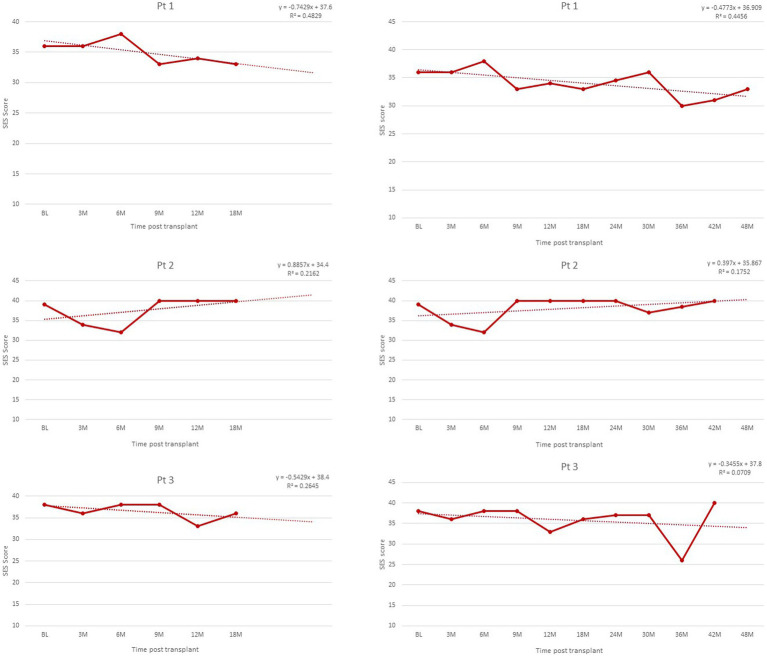
Self-esteem trajectories post-transplant, as measured by SES scores, range 10–40.

**Figure 4:**
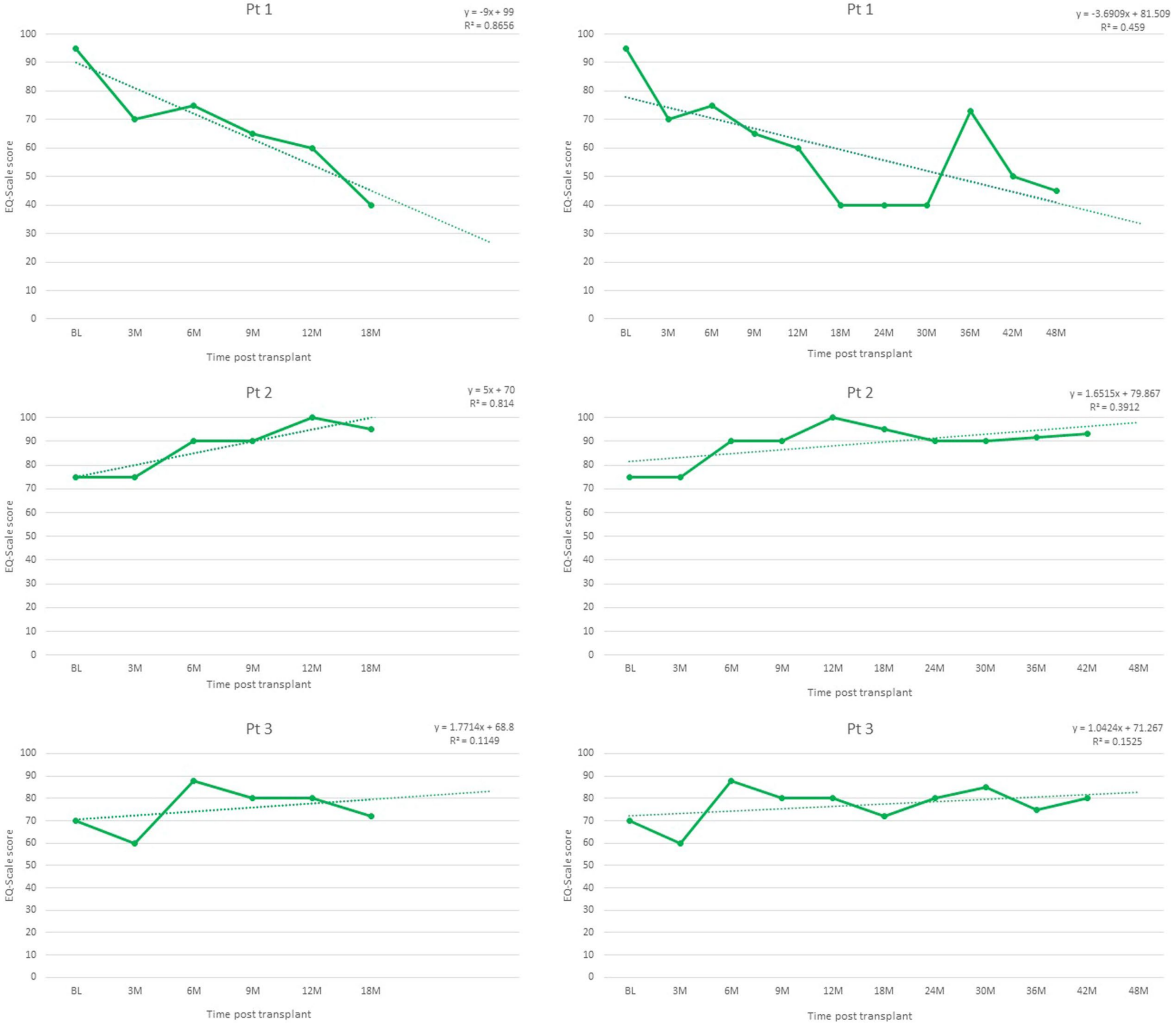
Quality of Life trajectories post-transplant, as measured by EQ-Scale scores, range 0–100.

Finally, outcome data was available to test the predicted trends in three of the patients at 24-, 30-, and 36-months post-transplant. Trends identified in all outcome trajectories were confirmed for all patients (Figures 2–4).

## Discussion

Our goal was to describe coping strategies associated with the most resilient mental health outcomes in this sample of face transplant recipients, pre-and post-transplant.

In line with the literature in other clinical populations, active coping strategies were associated with better mental health at baseline, while avoidant strategies showed strong associations with negative mental health. In our sample, poor baseline mental health appeared to be mostly linked to the use of avoidant strategies, such as denial, behavioral disengagement, and self-blame – which were strongly associated with poor outcomes in depression and self-esteem. The main clinical implication of this finding underscores the importance of assessing coping in face transplant candidates, so that interventions targeting ineffective strategies may be implemented to support the mental health of candidates while placed on a waitlist. Our findings also align with the generally held view that candidates able to leverage support-based strategies due to a strong support network are at an advantage in terms of pre-transplant mental health.

Post-transplant, we found that a different set of coping strategies predicted optimal outcomes. Both active and avoidant coping showed reversed patterns in mental health trends post-transplant compared to pre-transplant. This time, avoidant strategies were associated with better mental health outcomes in the 18 months following the transplant, in particular self-esteem – which may be boosted by ignoring negative experiences during a time of reduced agency in the face of rejection episodes and numerous medical follow-up procedures. Although intuitively surprising, a similar reversal pattern has been described in military studies, where the same coping strategy has been associated with opposite outcomes depending on military status. Namely, help-seeking was associated with negative outcome perception among active-duty Service Members, while it was associated with positive mental health outcomes in Veterans (Blais et al., 2014; Hom et al., 2017; Nazarov et al., 2020). It has been hypothesized that contextual factors, such as the fear of losing a security clearance while on active duty, may explain this reversal. Thus, contextual changes linked to the transition out of active duty similarly reversed the impact of the same coping strategy (Goode and Swift, 2019; Romero et al., 2020). In face transplant recipients, it is possible that the denial detrimental to seeking care pre-transplant played a protective role post-transplant. The loss of protective effect from support-based strategies may reflect a progressive deterioration of the support network of the recipients after years of intensive support, which should be controlled for in future studies when possible.

Although the coping strategies most associated with resilient mental health differed pre-and post-transplant in our sample, it is notable that trends in all mental health outcomes measured within the first 18 months post-transplant were confirmed in all patients up to 36 months post-transplant. This suggests that early mental health trajectories potentially offer valuable insight in longer term trends, thus highlighting the importance of an early monitoring of deteriorating mental health to propose supportive interventions.

## Clinical implications

Because face transplantation is a life-altering rather than lifesaving surgery, it is critical to select the candidates who stand to benefit most from the transplant in terms of psychosocial outcomes. In this sense, improving psychosocial outcomes is a close second goal to regaining function. The role of the face in our social identity, as much as its importance for both verbal and nonverbal communication through speech and facial expressions, make it one of the most salient physical contributors to our social functioning and interpersonal adjustment. Thus, medical teams have every mandate to improve the empirical markers based on which they can optimize surgical recommendations for face transplantation. Our findings suggest two main implications relevant to candidate selection and recipient care.

First, the discrepancy between coping styles most associated with positive outcomes pre-and post-transplant should offer a measure of caution regarding the protective value of pre-transplant active coping strategies. Rather, the best use of pre-transplant coping data may rest in informing therapeutic interventions at different phases of the transplant journey. When supporting candidates before the surgery, it may be beneficial to train candidates in non-avoidant strategies. Thus, rather than a one-size-fits-all coping recommendation, patients may be informed that optimal coping strategies may vary pre-and post-transplant, and encouraged to explore new coping strategies if experiencing poorer mental health post-transplant.

Second, and perhaps most promising, the finding that trends in mental health identified during the first 18 months post-transplant were maintained in all patients with available data and for all outcomes measured up to 36 months post-transplant suggests that early post-transplant data may provide reliable information to predict trends for the years following transplant, and valuable context to interpret the unavoidable ups and downs of punctual measurement. We propose that this finding supports the clinical recommendation to use early post-transplant data to guide case conceptualization and treatment planning when supporting the mental health of face transplant recipients post-transplant. To this effect, it may be relevant to increase the frequency of assessment during the first 6 months post-transplant, to propose therapeutic interventions as early as warranted.

## Limitations and future studies

As is still the norm in face-transplant studies, the small sample size limits the generalizability of these findings. Therefore, their value lies in both a thorough and novel description of available cases, and in outlining future research directions for the growing field of VCA. We can only encourage the replication of coping studies in other VCA cohorts to complement the descriptive findings reported here.

Another well-documented limitation in similar samples is the presence of a strong bias introduced by social desirability, or the desire to provide responses conforming to the perceived or imagined wishes of the medical team conducting the assessment. This is particularly true pre-transplant, during the candidacy phase, where candidates frequently report inflated self-esteem and underreport problem areas, such as substance use or lack of support. Our findings align with this effect, with a negative correlation between pre-and post-self-esteem scores, along with a strong correlation between avoidant styles (denial, disengagement) and self-esteem post-transplant, suggesting that self-esteem may be maintained by not looking too closely at difficulties.

Finally, it is notable that the three mental health measures did not correlate between pre-and post-transplant assessments. While depression showed only a minimal change, self-esteem and QoL both showed a moderately negative correlation with themselves, which could explain the reversal in pattern found in their association with specific coping strategies pre-and post-transplant. In future studies, assessing coping post-transplant could shed more light on this pattern by determining if patients have altered their coping style. Furthermore, the inclusion of other relevant psychosocial predictors, such as the occurrence of rejection episodes and life stressors, would contribute to a more granular understanding of mental health trajectories post-transplant.

## Conclusion

When it comes to helping medical teams with patient selection, only pre-transplant data is available to inform clinical decisions. Because it is the only data available, perhaps too much credit is given to its ability to predict post-transplant outcomes. In this important respect, the pattern of reversal we observed in our sample for the coping strategies most associated with resilient mental health pre-and post-transplant should caution against too strong a confidence in the maintenance of baseline patterns after the transplant. Instead, our findings support that early post-transplant data provides the most promising insight into longer lasting trends in the post-transplant mental health of face transplant recipients.

## Data availability statement

The datasets presented in this article are readily available by request to the senior author. Requests to access the datasets should be directed to bohdan.pomahac@yale.edu.

## Ethics statement

The studies involving human participants were reviewed and approved by Brigham and Women's Hospital IRB. The patients/participants provided their written informed consent to participate in this study.

## Author contributions

M-CN collected the data, conducted the data analysis, and wrote the manuscript. BP provided access to the patients and contributed to the manuscript. All authors contributed to the article and approved the submitted version.

## Funding

The Provost and Dartmouth College Library have provided the funds for open access publication fees of this manuscript.

## Conflict of interest

The authors declare that the research was conducted in the absence of any commercial or financial relationships that could be construed as a potential conflict of interest.

## Publisher’s note

All claims expressed in this article are solely those of the authors and do not necessarily represent those of their affiliated organizations, or those of the publisher, the editors and the reviewers. Any product that may be evaluated in this article, or claim that may be made by its manufacturer, is not guaranteed or endorsed by the publisher.

## Footnotes

^1^ https://local.psy.miami.edu/faculty/ccarver/sclBrCOPE.phtml
